# Facilitating Harvest of the Serratus Fascial Flap With Ultrasonic Dissection

**Published:** 2010-02-23

**Authors:** Iris A. Seitz, Craig Williams, Loren S. Schechter

**Affiliations:** ^a^Section of Plastic and Reconstructive Surgery, University of Chicago Medical Center, Chicago, Ill; ^b^Advocate Lutheran General Hospital, Park Ridge, Ill; ^c^Division of Plastic Surgery, Chicago Medical School, Rosalind Franklin University, Chicago, Ill

## Abstract

**Objectives:** Upper extremity reconstruction presents a functional and aesthetic challenge in plastic surgery. Exposure of vital structures often requires vascularized soft tissue coverage to achieve primary wound healing and optimize functional results. Specifically, the serratus fascial flap may satisfy the functional and cosmetic requirements for small- to medium-sized soft tissue defects of the upper extremity with limited donor site morbidity. We describe our technique of serratus fascial flap harvest, using the Harmonic SYNERGY curved blade (Ethicon Endo-Surgery, Cincinnati, Ohio). **Material and Methods:** A 21-year-old, right-hand-dominant, male carpenter and martial arts expert was involved in a motorcycle collision and sustained a left-hand dorsal degloving injury and extensor tendon rupture. Soft tissue reconstruction was performed with a serratus fascial free flap, immediate split-thickness skin graft, and palmaris longus tendon grafts. The flap was harvested with the Harmonic blade, which utilizes ultrasonic energy translated into mechanical energy, thereby allowing dissection and hemostasis simultaneously. **Results:** Flap elevation proceeded facilely using the Harmonic curved blade. The patient had no postoperative complications involving his flap or donor site. The closed suction drain in the donor site was removed on postoperative day 3, and the patient was discharged on postoperative day 10. The patient is doing well at 4 months follow-up. **Conclusion:** The Harmonic blade may assist in the dissection of the serratus fascial flap by aiding with hemostasis and minimizing surrounding tissue damage. This may reduce flap damage associated with harvesting techniques as well as decrease donor site seroma formation.

Upper extremity reconstruction following severe trauma is a challenge in reconstructive surgery. Soft tissue loss, combined with exposure of neurovascular structures, tendons, and/or skeletal structures, may further complicate the reconstructive effort. While the functional goals include restoration of a gliding surface for adequate tendon excursion, consideration should also be given to restoration of a cosmetically acceptable appearance. While multiple options for soft tissue coverage exist, flap choice depends on factors such as the location and size of the defect, associated injuries, patient comorbidities, donor site availability, and patient and surgeon preference.

Fascial flaps provide one option for thin, pliable, vascularized soft tissue coverage for small- to medium-sized upper extremity defects. Specifically, the serratus fascial flap may satisfy these requirements as well as offer the added benefit of minimal donor site morbidity.^1^ The serratus fascial flap has demonstrated excellent functional results in addition to acceptable aesthetic results without the need for additional debulking procedures.^2^ Furthermore, as the serratus muscle is not elevated from the chest wall, there is minimal donor site morbidity and pain. However, one potential drawback of this flap is the tedious and often bloody dissection of the serratus fascia from the underlying muscle. While standard flap elevation involves the use of bipolar cautery,^3^ we utilized the Harmonic blade in an attempt to facilitate flap harvest, while limiting thermal damage to the flap.

## MATERIALS AND METHODS

**Figure 1:**
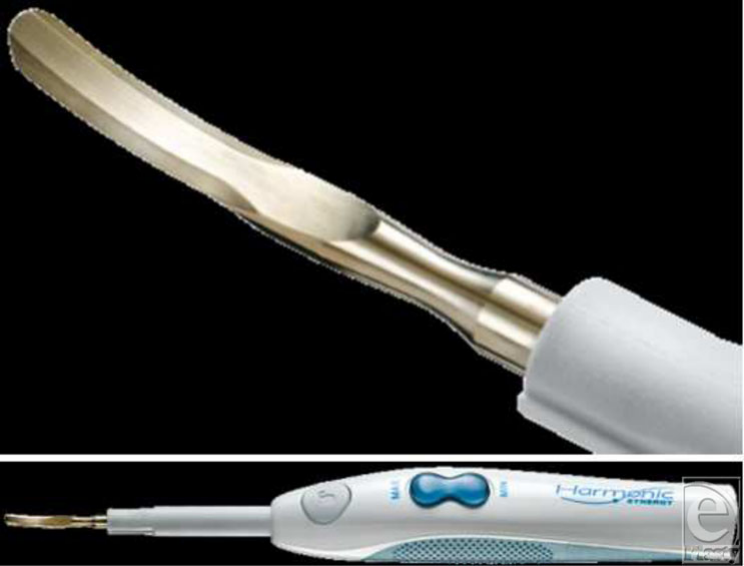

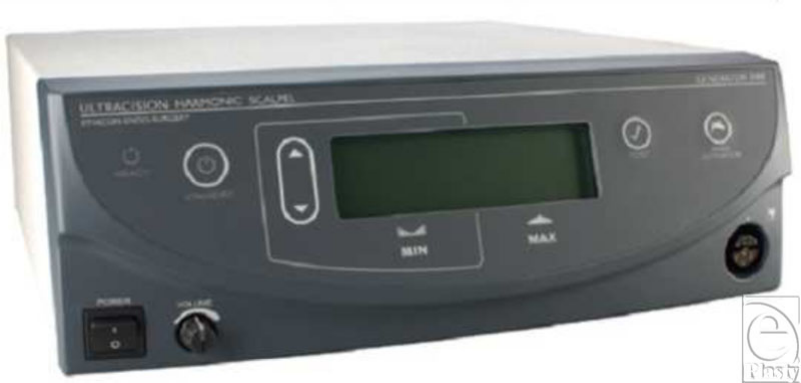
The Harmonic SYNERGY curved blade. The Harmonic generator with the hand piece and closeup view of the curved blade.

The Harmonic SYNERGY curved blade (Ethicon Endo-Surgery, Cincinnati, Ohio) (Fig 1) is a multifunctional instrument that converts high-frequency ultrasound waves (55.000 Hz/s) into mechanical energy, performing dissection and hemostasis simultaneously by disrupting hydrogen bonds and forming coagulum. The temperature generated is 80°C to 100°C compared with 200°C to 300°C through electrocoagulation. The ultrasonic vibration is transmitted from the transducer through an extending rod to the attached blade. The blade has a concave surface with a coagulating segment located posteriorly and cutting segments at the periphery.

## RESULTS

### Surgical technique

**Figure 2:**
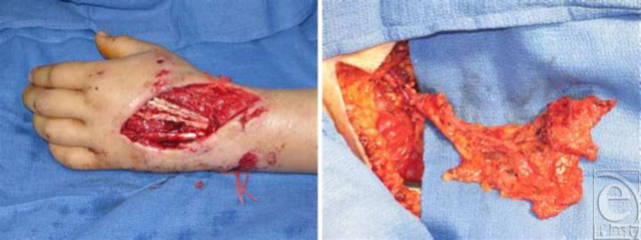
Intraoperative picture of patient's hand defect. Left: Defect of left dorsal hand after tendon repair. Right: Serratus fascial flap in situ attached to its pedicle after dissection using the Harmonic curved blade.

A 21-year-old, right-hand-dominant, male carpenter and martial arts expert was involved in a motorcycle collision and sustained a left-hand dorsal degloving injury and extensor tendon rupture. Initial management involved surgical debridement followed by negative pressure wound therapy for 1 week until the wound bed stabilized (Fig 2). Soft tissue reconstruction was performed with a serratus fascial free flap and immediate split-thickness skin graft. Tendon reconstruction was performed with palmaris longus tendon grafts.

Flap harvest proceeded through a 10-cm skin incision located along the anterior border of the latissimus dorsi muscle. The thoracodorsal pedicle was identified at its entrance into the latissimus muscle. At this point, the thoracodorsal pedicle was traced proximally to identify the serratus branch. A template of the defect was taken and used to design the flap in situ on the chest wall. The flap was elevated from the underlying serratus muscle in a distal to proximal fashion, and the surrounding loose areolar tissue was included with the flap. To facilitate flap harvest, the distal branches of the long thoracic nerve were included with the flap; however, the proximal branches were preserved to prevent scapular winging.

The elevation of the serratus fascia is usually accomplished with a fine bipolar forceps. In this case, the Harmonic blade was used to minimize thermal damage to the flap while simultaneously achieving hemostasis. By orienting the coagulating portion of the blade toward the muscle and away from the flap, the plane between the fascia and muscle was readily identifiable.

**Figure 3:**
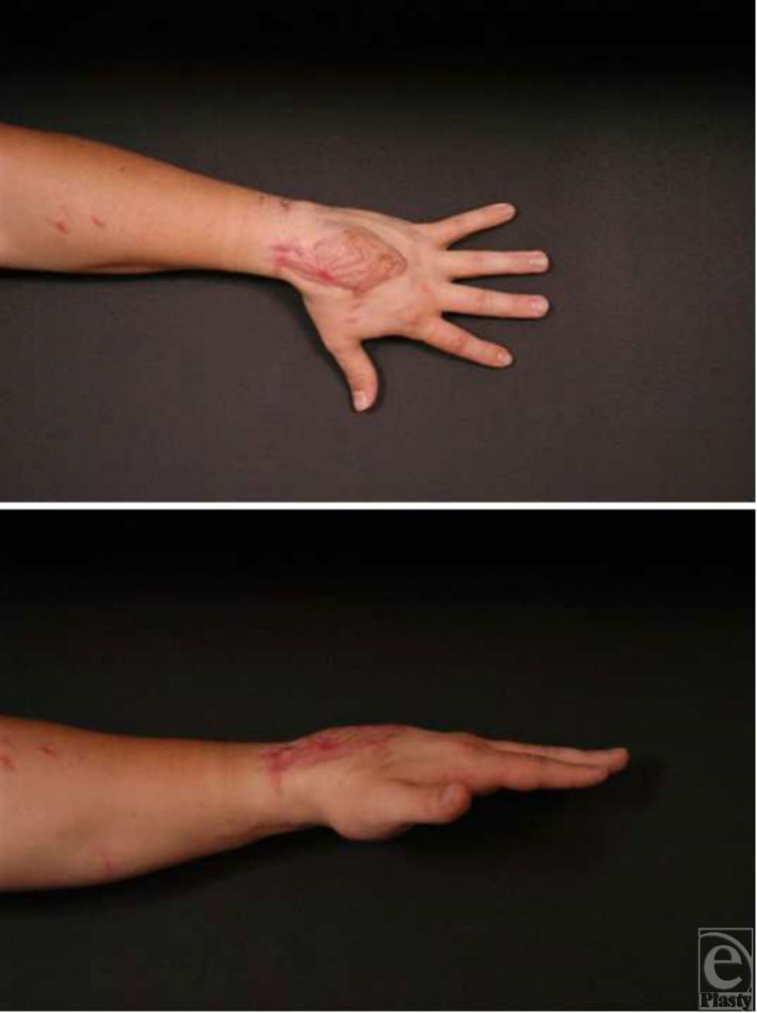
Patient at 4-month follow-up. The patient has good functional outcome and adequate contour restoration.

The patient had an uneventful postoperative course. The drain was removed from the donor site on postoperative day 3, and the patient was discharged on postoperative day 10. There were no complications and he regained full range of motion at 4-month follow-up (Fig 3).

## DISCUSSION

The serratus fascial flap provides vascularized soft tissue with limited donor site morbidity.^1^^-^4 However, one potential drawback of this flap is the dissection of the adherent serratus fascia from the underlying muscle. This dissection may be both tedious and bloody,^3^ and care must be taken to obtain adequate hemostasis without causing thermal injury to the flap. In some instances, immediate skin grafting of the transferred flap is deferred because of inadequate hemostasis. This may require several days of moist dressings or a temporary skin substitute until hemostasis of the flap is achieved.^3^

In our case, flap dissection was performed with the Harmonic blade. We believe that this not only facilitated dissection of the adherent serratus fascia from the underlying muscle but did so without causing thermal injury to the flap. The Harmonic blade can be used with the coagulating posterior aspect of the blade oriented toward the muscle and away from the fascial flap, thereby limiting injury to the flap. In addition, by elevating the flap with a single instrument, the surgeon may expedite flap dissection.

Furthermore, in a review of the plastic surgery literature, the Harmonic blade has been reported to decrease seroma formation in procedures such as latissimus dorsi and pectoralis major flaps as well as abdominoplasty procedures.^5^^-^7 Moreover, hemostasis and surrounding tissue damage seem to compare favorably to conventional electrocautery.^6^,^8^^-^10 In our institution, we also utilize the Harmonic scalpel to assist with harvest of omental free flaps. The Harmonic scalpel (ultrasonic shears) used in these procedures is designed slightly different from the curved blade used in this serratus fascial flap. This design facilitates coagulation of the short gastric vessels when dissecting the omentum from the greater curvature of the stomach. Once again, the use of a single instrument for flap dissection seems to reduce operative times. While a learning curve with this technology is expected, once comfortable, the surgeon may benefit from the reduced operative time.^6^,^10^

In this case report, we describe a novel use of the Harmonic blade. This case report is intended to introduce the potential benefit of ultrasonic flap dissection for a procedure known to be both rather tedious and potentially bloody. To quantify the potential advantages of the Harmonic blade over conventional techniques, a prospective study investigating blood loss, flap and donor site complications, and drain management is required. In addition, further experimental and clinical studies examining the effects of the Harmonic blade on tissue damage during flap surgery and the correlation with clinical outcomes will be beneficial.

## CONCLUSION

The Harmonic blade obtains hemostasis through the use of ultrasonic energy and may minimize surrounding tissue damage to both the flap and donor site. We found this technology to be useful in dissecting the adherent serratus fascia from the underlying serratus muscle, thereby allowing immediate skin grafting of the transferred flap.

**Financial disclosure:** No author involved in this work has any financial interest in any of the products or devices mentioned in this article.

## References

[1] Wintsch K, Helaly P Free flap of gliding tissue. J Reconstr Microsurg.

[2] Flügel A, Kehrer A, Heitmann C (2005). Coverage of soft-tissue defects of the hand with free fascial flaps. J Reconstr Microsurg.

[3] Meland NB, Weimar R (1991). Microsurgical reconstruction: experience with free fascia flaps. Ann Plast Surg.

[4] Ulrich D, Fuchs P, Bozkurt A Free serratus anterior fascia flap for reconstruction of hand and finger defects. Arch Orthop Trauma Surg.

[5] Ceccaldi PF, Ducarme G, Kéré D (2006). Effect of ultrasonic energy dissection technique in breast reconstruction with the autologous latissimus dorsi flap. J Gynecol Obstet Biol Reprod.

[6] Deo S, Hazarika S, Shukla NK (2005). A prospective randomized trial comparing Harmonic scalpel versus electrocautery for pectoralis major myocutaneous flap dissection. Plast Reconstr Surg.

[7] Richter DF, Zimman OA, Pérez JAL (2007). A randomized, prospective, parallel group study comparing the Harmonic™ to electrocautery in abdominolipectomies. Plast Reconstr Surg.

[8] Inaba H, Kaneko Y, Ohtsuka T (2000). Minimal damage during endoscopic latissimus dorsi muscle mobilization with the Harmonic scalpel. Ann Thorac Surg.

[9] Amaral JF (1995). Laparoscopic cholecystectomy in 200 consecutive patients using an ultrasonically activated scalpel. Surg Laparosc Endosc.

[10] Firmin FO, Marchac AC, Lotz NC (2009). Use of the Harmonic blade in face lifting: a report based on 420 operations. Plast Reconstr Surg.

